# Hospital Festivals Meetings, &c.

**Published:** 1895-11-02

**Authors:** 


### HOSPITAL FESTIVALS, MEETINGS, &C.

### THE YARROW HOME FOR CONVALESCENT CHILDREN, BROADSTAIRS.

It may be remembered that this home was opened for the reception of patients in July of this year, and on Saturday last arrangements were made by Mr. Yarrow, its founder, for members of the staffs of the various London hospitals, and others interested in such matters, to inspect the home in working order. Some 250 visitors went down by special train from Victoria to Broadstairs on Saturday, and none could have failed to admire the handsome building on its splendid site upon the Downs above Broadstairs, or to appreciate the generous manner in which Mr. Yarrow has carried out his good work. The Yarrow Convalescent Home has been fully endowed, and no pecuniary help is needed or desired. It contains 100 beds—for 50 boys and 50 girls—and is intended, not for the children of the very poor, but for those of a superior class whose parents are in such reduced circumstances as to render it impossible for them to send their children, after illness, for a necessary change to the sea entirely at their own cost. The parents are expected to pay 5s. a week towards the maintenance of a child, in addition to the railway fare, which by arrangement with the London, Chatham, and Dover Railway Company is 1s. 9d. each way between London and Broadstairs. Children between the ages of 4 and 16 years are admitted, irrespective of nationality or creed, provided their home training has been such as to make them fit companions for other respectable and well-mannered children, and who have not been suffering from any infectious disease. Such an institution for the benefit of the large and struggling class above the very poor, for whom many charities already exist, has been much needed, and Mr. Yarrow's endeavour to meet this need should meet with ready recognition and appreciation. Full particulars with regard to terms of admission may be had from the Secretary, at the London office, 73A, Queen Victoria Street.

